# Exploring the Non-Invasive Approaches to Carpal Tunnel Syndrome in Routine Clinical Practice: A Focus on the Role of Acetylcholinesterase Inhibitors

**DOI:** 10.3390/medicina60081219

**Published:** 2024-07-27

**Authors:** Ojārs Rubens, Solvita Bērziņa, Anda Rozenbaha, Guna Dansone, Yulia Troshina

**Affiliations:** 1 Pauls Stradins Clinical University Hospital, LV-1002 Riga, Latvia; 2 Neuro LLC, LV-1039 Riga, Latvia; 3 Medical and Clinical Research Department, JSC Olpha, LV-2114 Olaine, Latvia

**Keywords:** carpal canal, conservative treatment, acetylcholinesterase inhibitor, peripheral neuropathy

## Abstract

The prevalence of *N. medianus* compression neuropathies remains high in clinical practice. The objective was to evaluate modalities of conservative treatments for carpal tunnel syndrome (CTS) focusing on the role of acetylcholinesterase inhibitors. This observational study involved 51 adult outpatients diagnosed with CTS. Patients were observed during routine clinical protocols and we compared two groups of 25 and 26 individuals, with the first group receiving basic therapy for CTS and 20 mg of ipidacrine (Neiromidin^®^) two or three times a day per os, while the second group received only basic therapy. The condition of all patients was assessed twice, with at least a one-month interval. The parameters evaluated included the Boston Carpal Tunnel Questionnaire (BCTQ); the Disabilities of the Arm, Shoulder, and Hand scale (DASH); and pain intensity on the Numeric Rating Scale (NRS). The mean reduction in DASH score was 12.3 (SD 7.7) in Group 1 and 7.1 (SD 6.3) in Group 2 (*p* < 0.01). Also, other scores showed statistically significant differences between the two groups: −2.3 vs. −1.0 for NRS, −0.89 vs. −0.44 for SSS, and −0.68 vs. −0.31 for FSS, respectively (*p* < 0.01). Moreover, these findings correlated positively with the global improvement (CGI-I) between the groups. The addition of ipidacrine to basic therapy led to improved recovery in patients with CTSs of varying severity.

## 1. Introduction

Carpal tunnel syndrome (CTS) is a commonly diagnosed disorder of the peripheral nervous system and often manifests as a chronic condition that significantly reduces the quality of life, especially among the working population. CTS frequently occurs in patients with diabetes or obesity and in individuals whose occupation involves repetitive hand movements [1,2]. Women are more susceptible to developing CTS than men, at a ratio of 2.2:1 [3]. Notably, around 22% of office workers suffer from wrist pain and numbness, significantly reducing their productivity, and the average number of days an employee may miss work due to CTS can reach 27 or higher [4,5].

Currently, CTS treatments are limited to the general recommendations of ergonomic practices, hand orthosis, glucocorticoid therapy, and surgery, in severe cases. There is also evidence of electrotherapy having some efficacy. Surgical therapy is generally only recommended if conservative treatment fails or in severe cases of the disease [6].

Therefore, the search for a drug that can positively influence nerve fiber recovery and improve motor and sensory functions remains relevant.

Ipidacrine, a reversible inhibitor of acetylcholinesterase, registered under the trade name Neiromidin^®^, is authorized in Latvia and several other countries inside and outside the European Union for the treatment of peripheral and central nervous system conditions involving nerve damage and impulse transmission disorders. Ipidacrine affects acetylcholinesterase levels due to its ability to block sodium and potassium channels [7,8]. This leads to increased action potential, conductivity, and moderate analgesic effects, making it a good candidate for the treatment of peripheral neuropathies such CTS. Neiromidin tablets that are 20 mg are widely prescribed in ambulatory practice.

We have undertaken an attempt to evaluate the role and potential of ipidacrine specifically in the therapy of median nerve neuropathy. The high incidence and prevalence of CTS in general population, coupled with the significant personal and socioeconomic costs associated with the given condition, underscore the necessity of this research.

### Objective

The exploratory objective of this work was to estimate the potential role of the acetylcholinesterase inhibitor ipidacrine in the treatment of median nerve compression and to gather preliminary information in support of further research hypotheses.

## 2. Materials and Methods

A non-interventional observational study was performed at a neurologist’s practice between July 2018 and July 2019. In total, 57 protocol-compliant adult outpatients receiving conservative treatment for grade 1–3 CTS [9] were identified in the practice’s medical records. The main criteria included men and women aged 18–65 years with a clinically and electroneurographically confirmed diagnosis of CTS, BCTQ scores > 1 on a Likert scale (summary points SSS > 11 and FSS > 8), and no therapy with ipidacrine or other cholinesterase inhibitors in the month before their inclusion in the study. The exclusion criteria ruled out individuals with significant damage to multiple peripheral nerves (including those of traumatic origin), those within a post-surgery period, those who had received recent corticosteroid injections, those with the presence of the wrist masses, and patients suffering from a severe somatic and/or cognitive impairment that might potentially interact with the study outcomes. The research involved two assessments with at least a one-month interval to compare the participants’ condition at baseline and at follow-up. Six participants did not return for the follow-up; consequently, fifty-one individuals who fully met the protocol criteria were included in the statistical analysis. Before commencing, the study was reviewed and approved by an Ethics Committee. All participants provided consent for the use of their medical data. The 51 participants that were included in the study analysis (7 men and 44 women, reflecting the global gender split of cases of CTS [10]) were statistically similar in age and gender between the groups. Both groups received basic therapy (pharmacologic and non-pharmacologic), with Group 1 additionally receiving ipidacrine at a dosage of 20 mg (1 tablet) two or three times a day per os. Both at baseline and follow-up, subjects underwent examinations using validated scales—the Boston Carpal Tunnel Questionnaire (BCTQ); the Hand, Wrist, and Shoulder Disability Scale (DASH); and pain intensity on the Numeric Rating Scale (NRS)—along with a comprehensive clinical examination and neurological status assessment.

The BCTSQ is designed specifically for individuals with CTS to assess their symptoms and severity of their symptoms when performing specific actions. It consists of two separate scales: the Symptom Severity Scale (SSS), which contains 11 items, and the Functional Status Scale (FSS), which contains 8 items. Respondents score the difficulty of each item on a 5-point Likert scale for 24 h periods over the past 2 weeks. A final score (the sum of the individual scores divided by the number of items) is calculated for each scale and ranges from 1 to 5, with a higher score indicating a more severe disability. The BCTQ is a valid and reliable tool for primary outcome measures in CTS studies. The DASH is a 30-item questionnaire to rate difficulty and interference with daily life; it has been demonstrated to be a valid and reliable questionnaire for a variety of upper extremity musculoskeletal disorders. The DASH score ranges from 0 (no disability) to 100 (the most severe disability). The NRS is a commonly used tool to assess pain severity at the moment in time using a 0–10 scale, with zero meaning “no pain” and 10 meaning “the worst pain imaginable” [11,12,13].

Nerve provocation tests (Durkan’s, Tinel’s, Phalen’s, reverse Phalen’s, hand elevation tests) and a nerve conduction study (electroneurography) were conducted. A General Clinical Impression (CGI-I) took place at follow-up. Data processing was performed using non-parametric methods—a Wilcoxon test and Mann–Whitney U test—and IBM^®^ SPSS Statistics, v22. All tests were two-sided and performed at a 5% significance level. The results were presented using medians and means with standard deviations.

## 3. Results

### 3.1. Patients’ Baseline Characteristics

The patients’ baseline characteristics (Table 1) included age, sex, CTS parameters (severity, NRS, BCTQ), and DASH score. In total, 44 females and 7 males aged from 29 to 64 years old were included in the statistical analysis. The mean age of the patients was 53 ± 9.6 years old in Group 1 and 47 ± 10 years in Group 2; in both groups the majority were women (84% and 89%, respectively), but the gender proportion within groups remained similar. Out of the 51 patients, 35 (69%) had bilateral CTS, with higher presence of unilateral CTS in Group 2. At baseline, the patients in Group 1 were slightly older and had more severe CTS and disability levels compared to Group 2.

At follow-up, a significant difference was observed between the groups in terms of the change in NRS, DASH, and BCTQ scores, demonstrating a more prominent positive dynamic in Group 1 (Table 2). A Minimal Clinically Important Difference [11,12,13] was observed in both groups for FSS, but only in Group 1 for the rest. The findings were in line with a clinician’s assessment of improvement: the mean CGI-I in Group 1 was 2 (1.8 ± 1.0), “much improved”, while it was 3 (2.8 ± 1.5), “minimally improved”, in Group 2 (*p* = 0.01).

Despite the dispersed individual number of days between the two assessments, the mean interval was similar in Group 1 and Group 2 (70 ± 14 days and 73 ± 18 days, respectively; *p* = 0.54). Moreover, a correlation between the number of days between assessments and the change in self-reported patient outcomes shown in Table 2 was not observed. A moderate negative correlation (0.3–0.4, *p* = 0.05) was observed between CTS severity at baseline and the given outcomes, suggesting somewhat less pronounced positive dynamics in patients with more severe CTS. The correlation between the change in patient-related outcomes in the whole study population remained strong (0.6–0.7, *p* = 0.01), addressing potential concerns about intrasubject variation.

### 3.2. CTS Therapy/Concomitant Treatments

Patients were observed in routine clinical practice under standard treatment protocols, including both nonpharmacological and pharmacological methods: ergotherapy, hand orthoses, physical therapy, stretching, acupuncture, kinesiology tape, massage, NSAIDs, analgesics, anticonvulsants, B group vitamins, and acetylcholinesterase inhibitors (20 mg ipidacrine tablets) were administered to Group 1. In general, the patients in Group 1 were prone to receive more treatment modalities than patients in Group 2, with statistically significant difference in terms of non-pharmacological methods (Table 3); however, subsequent effects could not be properly evaluated due to the observed heterogeneity of treatment combinations and regimens between the individuals.

Compliance with the 20 mg ipidacrine tablets treatment was checked at follow-up and reported as good for all patients in Group 1.

### 3.3. Monitoring of N. Medianus Function

**Figure 1:**
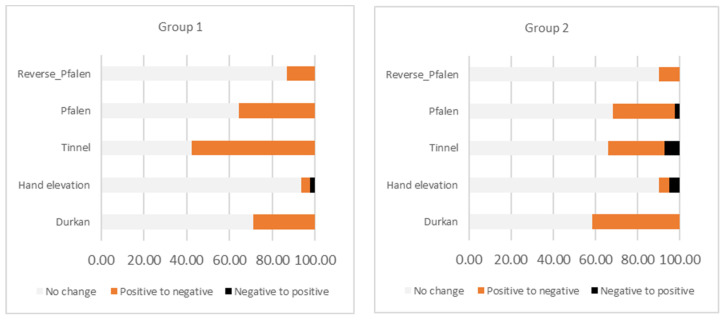
Nerve provocation test results for groups—baseline vs. follow-up.

The function of *N. medianus* was assessed using provocative tests, the dynamics of which are presented in Figure 1. Notably, substantial changes (the number of transitions from a positive to a negative sign for specific symptoms by groups) were observed across all tests. However, the most significant and specific response was evident in the Phalen, Tinnel, and Durkan tests, with the regression being most pronounced in both groups. Additionally, in Group 2, three out of five tests demonstrated deterioration (reversal from negative to positive), indicating a worsening of conditions in some subjects in this group, whereas, in the ipidacrine group, this occurrence was noted in only 2.2% of cases (compared to 14.6% cumulatively for the other group).

**Figure 2:**
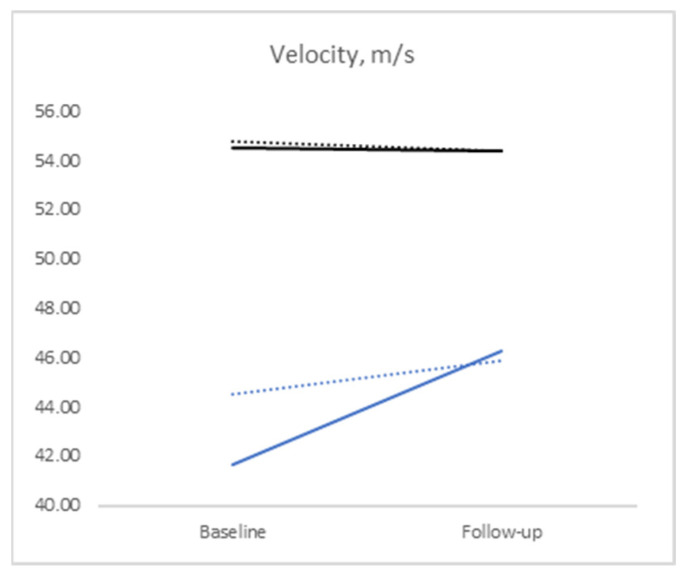

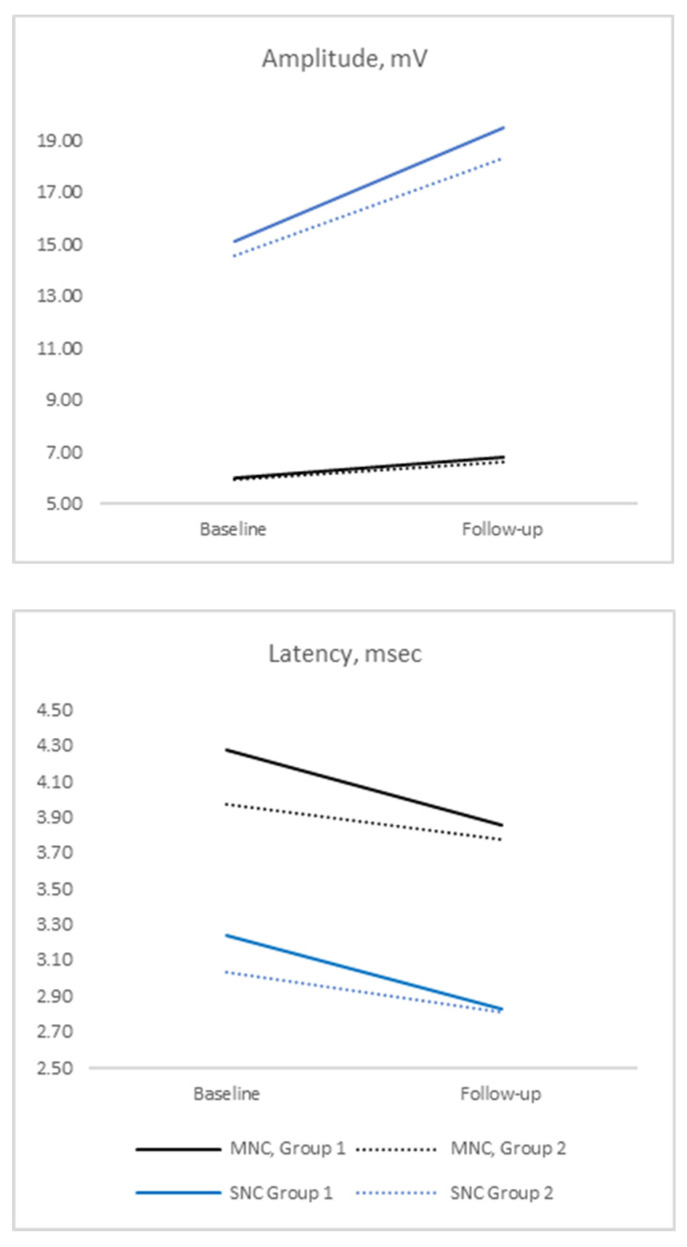
Motor and sensory nerve conduction (MNC, SNC) parameters remained relatively unchanged (velocity and signal amplitude for motor fibers) or virtually improved in both groups from baseline to follow-up. In Group 1, the conduction velocity of the sensory fibers, and their signal latency, increased nearly to the lower limit of normal.

Furthermore, electroneurographic indicators (conduction velocity (m/s), amplitude (mV), and latency (ms)) were evaluated for the motor and sensory fibers (Figure 2). The conduction velocity of the motor fibers remained relatively unchanged from the baseline level in both groups and approached physiologically normal values. In contrast, impulse propagation along the sensory fibers was notably hindered at the baseline level (and worse in the Group 1), significantly increasing almost to the lower limit of normal in the ipidacrine group and remaining virtually unchanged in Group 2. The amplitude of the signal revealed a contrasting pattern, affecting more sensory fibers in both groups, while the motor indicator remained nearly unchanged. Signal latency showed a significant reduction in all groups, but only in the ipidacrine group (Group 1) did the indicators practically approach normal limits at the follow-up.

## 4. Discussion

The persisting prevalence of carpal tunnel syndrome coupled with the moderate effectiveness of the available conservative approaches, which are, though, routinely practiced as the first treatment step, especially for mild to moderate CTS, underscores the current medical goal of discovering and utilizing new pathogenetically acting drugs [14,15,16]. One such noteworthy medication is ipidacrine, which reversibly inhibits acetylcholinesterase and influences potassium and sodium channels, thereby enhancing conductivity and action potential height and providing a moderate analgesic effect. Its relatively short half-life prevents its accumulation in the body, allowing for the precise targeting of conductivity and peripheral nerve sensitivity [7]. This was demonstrated in the aforementioned study, where a deliberately more severe group of carpal tunnel syndrome patients receiving ipidacrine therapy exhibited better outcomes compared to the group not receiving ipidacrine, both objectively (ENG, especially sensory responses) and subjectively (provocative tests and self-assessment scales). It is noteworthy that the group receiving ipidacrine virtually lacked an increase in the severity of their provocative tests (observed in only 2.2% of cases), suggesting a halt in neurodegenerative processes—in contrast, the other group demonstrated around a 15% increase in test manifestations.

This study has several limitations. Patients were observed during routine clinical practice under standard, yet different, treatment protocols; the nonpharmacological methods and pharmacological classes of the drugs, as well as their duration of administration and criteria of use, were not unified, thus their proper effects on efficacy could not be fully addressed. There were not sufficient data to evaluate the impact of patients’ daily activity or occupational factors during their treatment. Finally, long-term outcomes remain out of the scope of this study.

## 5. Conclusions

Acetylcholinesterase inhibitors, such as ipidacrine, could serve as a valuable addition to the basic therapy for carpal tunnel syndrome. It is suggested that these substances selectively stimulate impulse conduction and its amplitude, facilitating recovery and rehabilitation in patients with CTS. ENG monitoring did not reveal any adverse trends in nerve condition parameters among the study population. This observational study provides a sound justification for further well-designed interventional clinical studies aimed at providing additional safe and effective tools for the management of peripheral nervous system disorders such as carpal tunnel syndrome.

## Figures and Tables

**Table 1:** Patients’ baseline characteristics.

Parameter	Group 1 N = 25	Group 2 N = 26	*p*-Value
Age, years *	53.2 (9.6)	47.3 (10)	0.05
Sex, M/F, n (%)	4/21 (16/84)	3/23 (11.5/88.5)	-
Unilateral/bilateral CTS, n (%)	5/20 (20/80)	11/15 (42.3/57.7)	-
CTS grade #† Grade 1, % Grade 2, % Grade 3, %	3 (3–2) 4 28 68	2 (2.25–2) 3.8 73.1 23.1	<0.01
NRS *	4.7 (1.5)	3.7 (1.4)	<0.01
BCTQ:			
SSS *	2.6 (0.6)	2.1 (0.5)	<0.01
FSS ^*	2.1 (0.6)	1.8 (0.5)	0.03
DASH *	23.1 (9.6)	18 (9.9)	0.03

* Mean (SD). † Median (IQR). # Most severe grade for patients with bilateral CTS. ^ Dominant hand only.

**Table 2:** Change in self-reported patient outcomes from baseline.

Assessment (Mean, SD)	Group 1 N = 25	Group 2 N = 26
NRS	−2.27 (1.6) *	−1.0 (1.7)
BCTQ:		
SSS	−0.89 (0.6) *	−0.44 (0.3)
FSS ^	−0.68 (0.5) *	−0.31 (0.31)
DASH	−12.3 (7.7) *	−7.1 (6.3)

^ Dominant hand only, * *p*-value < 0.01.

**Table 3:** Quantitative assessment of concomitant treatments.

Treatment Methods Received	Group 1 N = 25	Group 2 N = 26	*p*-Value
Pharmacological *, n (SD)	2.9 (1.0)	2.4 (1.5)	0.29
Min-Max, n	0–4	0–5	-
Non-pharmacological, n (SD)	5 (1.9)	4.5 (1.5)	0.03
Min-Max, n	0–7	2–7	-

* Excluding ipidacrine.

## Data Availability

The anonymized data presented in this study are available on request from the corresponding author.
